# Acute Respiratory Distress in a Pediatric Patient With Prader-Willi and Moebius Syndromes

**DOI:** 10.7759/cureus.29335

**Published:** 2022-09-19

**Authors:** Jamie Thomas, Taylor Butts, Jason Burtch, Natalie F Smith, Pooja Kethireddy, Jenny Gutwein, Cristina Figallo-Cuenca

**Affiliations:** 1 Osteopathic Medicine, Nova Southeastern University Dr. Kiran C. Patel College of Osteopathic Medicine, Fort Lauderdale, USA; 2 Neurology, Nova Southeastern University Dr. Kiran C. Patel College of Osteopathic Medicine, Fort Lauderdale, USA; 3 Medicine, Nova Southeastern University Dr. Kiran C. Patel College of Osteopathic Medicine, Fort Lauderdale, USA; 4 Pediatrics, Broward Health Medical Center, Fort Lauderdale, USA

**Keywords:** respiratory distress, dornase alfa, congenital disease, neonatal hypotonia, severe dyspnea, moebius sequence, prader-willi syndrome

## Abstract

Although acute respiratory infections or diseases such as asthma commonly cause respiratory distress in a pediatric patient, neuromuscular disorders must be considered as a possible etiology in patients with significant hypotonia, neurological deficits, and gross developmental delay. We present a case where a patient’s lack of response to initial asthma exacerbation therapy led to a reconsideration of the original diagnosis and adaptation of the management plan. Our patient presented with a rare combination of two congenital disorders that cause hypotonia: Prader-Willi syndrome and Moebius syndrome. This case underlines the importance of considering atypical etiologies in pediatric patients with respiratory distress, while also illustrating the effectiveness of the atypical use of Dornase alfa in a patient with underlying neuromuscular disorders.

## Introduction

Although frequently caused by acute respiratory infections or diseases such as asthma, respiratory distress in pediatric patients can present secondary to a myriad of possible etiologies, such as congenital hypotonic syndromes. Prader-Willi syndrome (PWS) is a relatively rare disease caused primarily by a deletion of the paternally active genes on chromosome 15, which leads to a variety of clinical features including early-onset obesity, hypotonia in infancy, hyperphagia, and global developmental delay [1]. Moebius syndrome (MBS) is a rare congenital dysinnervation palsy that affects cranial nerves (CN), most commonly the abducens (CN VI) and facial (CN VII) nerves; however, other nerves such as CN VIII, IX, X, XII have also been reported [2]. This syndrome may have a unilateral or bilateral presentation and tends to be diagnosed early with symptoms such as a weak suck (CN VII) or poor tracking (CN VI) [3]. We present a unique case with atypical management of a female infant diagnosed with both PWS and MBS, a rare combination that is lacking representation in literature.

## Case presentation

We present a case of a 23-month-old female presenting to the Emergency Department (ED) of a local hospital with two days of non-bloody, non-bilious emesis, noisy breathing, and shortness of breath. The patient also had an associated fever, productive cough with clear sputum, and nasal congestion for two days. According to her mother at the bedside, the patient was diagnosed with asthma, PWS, and MBS in Chile; however, she was unable to provide prior medical documentation. The patient was born prematurely at 34 weeks of gestation via Cesarean section. Her past surgical history included the placement of a gastrostomy tube due to her inability to feed. She also had a Nissen fundoplication in which the upper part of the stomach is wrapped tightly around the lower part of the esophagus to help prevent severe reflux in her neonatal period. She was currently taking Albuterol as needed and Fluticasone twice daily via a nebulizer and was up to date with her vaccinations.

**Figure 1:**
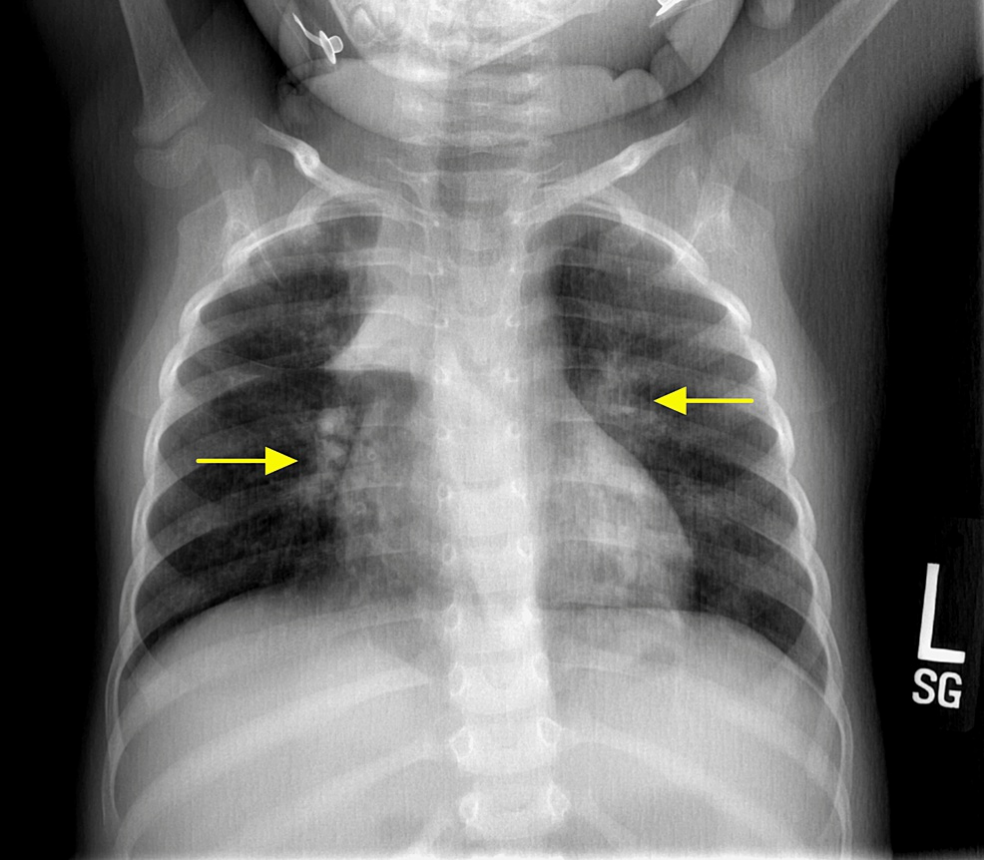
Patient's CXR showing interstitial prominence in the perihilar regions, indicated by the arrows.

Upon presentation to the ED, the patient was in acute respiratory distress with an oxygen saturation of 95% and had diffuse bilateral rhonchi. Her chest x-ray (CXR) showed interstitial prominence in the perihilar regions. Due to her past medical history and presenting symptoms, asthma and a viral illness were considered her top two differential diagnoses at the moment, although other possible etiologies were still being considered (Figure 1). Her respiratory viral panel was negative and her complete metabolic panel, complete blood count, urinalysis, urine and blood culture were all unremarkable. Due to her acute respiratory distress in the ED, the patient was initially suspected to have an acute asthma exacerbation and was treated accordingly in an attempt to rapidly resolve her acute distress. She was initiated on oxygen via nasal cannula and received three doses of Albuterol-Ipratropium, one normal saline bolus, one dose of Methylprednisolone, and Racemic Epinephrine, as well as Ondansetron for nausea and Ibuprofen for fever. However, despite treatment, her respiratory distress continued to worsen.

**Figure 2:**
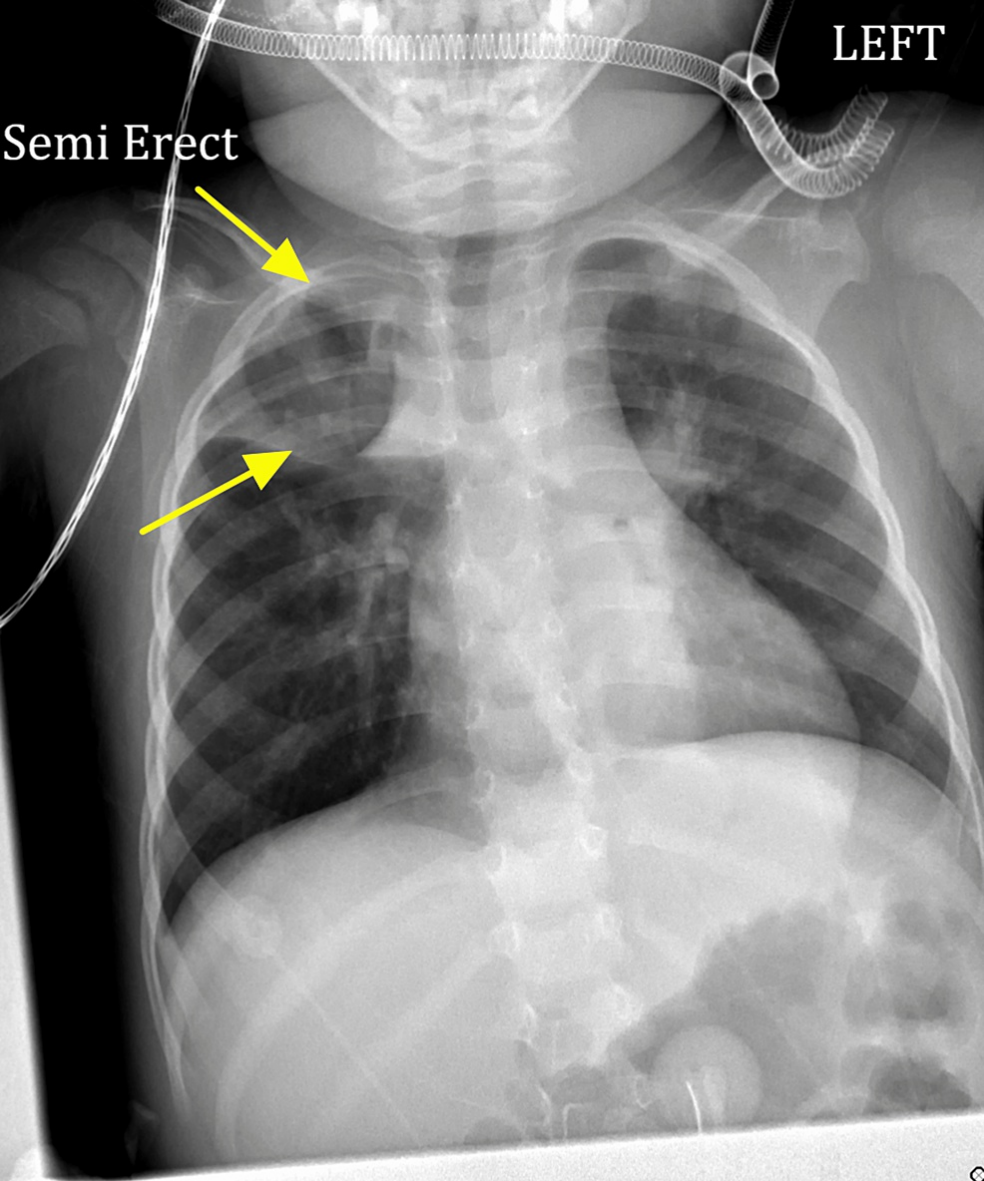
Patient's CXR showing right upper lobe opacities likely indicative of atelectasis or pneumonia, indicated by the arrows.

Two days after admission, the patient’s physical exam was unchanged, and a repeat CXR showed right upper lobe opacities likely indicative of atelectasis or pneumonia (Figure 2). However, since the laboratory exams showed no signs of leukocytosis or bandemia, and urinalysis was unremarkable, antibiotics were held at this time. A failure to show any signs of improvement prompted the discontinuation of the bronchodilators. The focus was shifted to consider other differentials due to her prior diagnoses of PWS and MBS, which both increase her risk of possible aspiration secondary to hypotonia.

A neurologically focused physical exam was conducted to see if the patient’s previous diagnoses of PWS and MBS were accurate. The patient met the diagnostic criteria of five points for PWS (Table 1) [4]. She presented with major criteria features including micrognathia; global developmental delay as she was unable to sit up on her own and did not reach for objects; diffuse hypotonia; and continued feeding difficulties. In terms of minor criteria for PWS, the patient presented with extreme lethargy, had vision abnormalities as she was unable to track with her eyes and failed to respond to visual stimulation, and had speech defects.

**Table 1:** Major and minor criteria in the diagnosis of Prader-Willi Syndrome. Score one point for each major criterion and 0.5 point for each minor criterion. A diagnosis of PWS should be suspected in children younger than three years of age with a score of at least 5; and in children aged three years and older with a score of at least 8, with 4 points from the major criteria.

Major Criteria	Minor Criteria
Characteristic facial features (includes almond-shaped eyes, down-turned mouth, narrow bifrontal diameter, strabismus, thin upper lip).	Decreased fetal movement and infantile lethargy
Developmental delay.	Esotropia, myopia.
Feeding problems/failure to thrive during infancy.	Hypopigmentation.
Hypogonadism (may include cryptorchidism, hypoplastic scrotum, small testes, hypoplastic labia minora and clitoris, and delayed puberty).	Narrow hands with straight ulnar border.
Infantile hypotonia.	Short statue (compared with family members).
Rapid weight gain between one and six years of age.	Skin picking.
	Sleep disturbance/sleep apnea
	Small hands and feet.
	Speech articulation defects.
	Thick, viscous saliva.
	Typical behavioral problems.

Our patient also presented with multiple characteristics commonly present in MBS (Table 2) [3]. She had feeding difficulties caused by poor sucking secondary to incomplete closure of her lips leading to the need for a gastrostomy tube. She also had an inability to completely close her eyes while sleeping, a fixed gaze characterized by failure to track, a language/speech delay in terms of not producing any words by 23 months old, limited jaw opening, micrognathia, and generalized hypotonia.

**Table 2:** Moebius syndrome signs secondary to cranial nerve involvement.

Area	Feeding and oral area	Percentage	Language and hearing	Percentage	Vision/visual	Percentage
Cranial nerve involvement	V, VII, IX, XI, XII		VII, VIII, IX, X, XI, XII		III, IV, VI, VII	
Functional Deficit	Poor neonatal sucking and swallowing	37.8	Hearing loss	6.8	Ocular motor deficit	89.8
	Need for nasogastric tubes and gastrostomy	5.5	Language delay	31	Visual deficit	19.3
	Nutritional problems	16	Speech deficit	42	Photophobia	15
	Dental problems	17				
	Palatal problems and micrognathia	7.4				

Due to the patient’s hypotonia, the possibility of impaired airway clearance with increased secretions secondary to underlying neuromuscular weakness and subsequent anatomical upper airway compromise was then considered as the etiology of the patient’s presenting symptoms. Pulmonology was consulted and prescribed Dornase Alfa 2.5 mg and pulmonary hygiene with hypertonic saline nasal suctions. The patient immediately showed signs of improvement in her respiratory distress.

**Figure 3:**
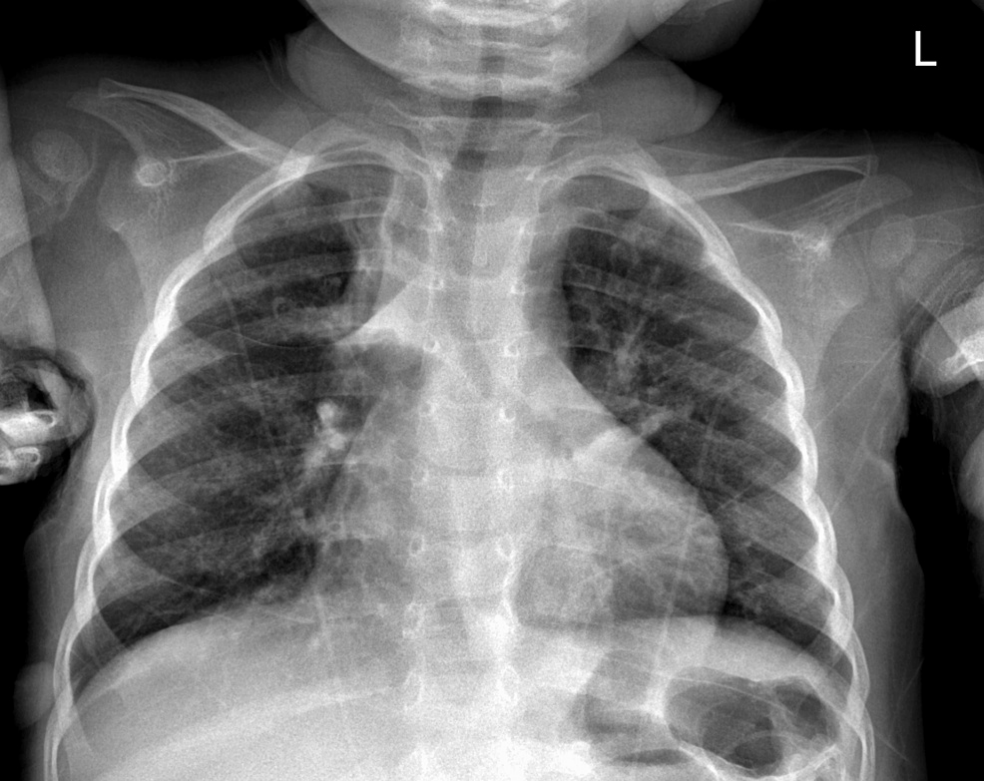
Patient's CXR demonstrating improved aeration of the lungs.

The patient continued to improve over the next three days, and a subsequent physical exam showed that the rhonchi and tachypnea had resolved. A repeat CXR also demonstrated improved aeration of the lungs (Figure 3). The patient was discharged with her previous home medications of Albuterol and Fluticasone to manage her asthma and Famotidine to manage her reflux. The patient's mother was advised to follow up with her outpatient Pediatrician, Gastroenterologist, Pulmonologist, and Otorhinolaryngologist.

## Discussion

Respiratory distress, characterized by difficult and labored breathing, is a common symptom seen in about 25% of patients in the ambulatory setting and comprises about 7.4% of patients presenting to emergency rooms [5]. Neurological causes of respiratory distress must be considered when approaching a pediatric patient with significant hypotonia, neurological deficits, and gross developmental delay. Potential etiologies including congenital myopathies, congenital muscular dystrophies, congenital myasthenic syndromes, metabolic myopathies, spinal muscular atrophy, PWS, and MBS should be considered [6]. Each of these diagnoses is rare, with PWS having a birth incidence of 1/10,000-1/30,000 and MBS having a birth incidence of 1/250,000. To our knowledge, this is the first case report in the literature that presents both. 

The diagnosis of PWS involves both clinical suspicion and genetic testing [7]. As shown previously (Table 1), the major and minor criteria can aid in the confirmation of the diagnosis. However, genetically, deletion of the paternal 15q11.2-13 is the defect that accounts for the majority of PWS cases. These alleles from the maternal chromosome 15 are imprinted through hyper-methylation and are therefore silenced. The lack of expression of the paternally inherited genes occurs by three mechanisms, deletion of the 5-6Mb region from the paternal chromosome 15 (65%-75%), maternal uni-parental disomy (20%-30%), and an imprinting defect (1%-3%) [8].

Unlike PWS, MBS is diagnosed solely based on clinical criteria. It is a rare neurological disease that is primarily characterized by non-progressive uni- or bi-lateral CN palsies, specifically the abducens (CN VI) and facial (CN VII) nerves, which affect lateral eye movement as well as facial expression [9]. CN VII is the most commonly affected nerve, present in about 96% of the cases, and CN VI in 85% of the cases [3]. Although less common, studies have demonstrated that other cranial nerves may be affected as well, including CN VIII, IX, X, and XII [2]. Due to dysfunction in multiple CNs, patients often present with a variety of motor, feeding, language, and visual impairments.

MBS can be diagnosed as early as the neonatal period. These patients commonly present with a lack of facial mimicking, poor or absent sucking due to incomplete closure of the lips, fixed gaze, incomplete eyelid closure during sleep, and ptosis. In a recent study, patients with MBS were analyzed and the primary motor problems observed consisted of hypotonia, sucking-swallowing difficulty, breathing problems, and dysphagia that may result in a gastrostomy. It was found that about 31% of the patients had a language delay and 42% had a speech deficit [3].

Based on her previous history of asthma, the initially presumed etiology of our patient’s acute respiratory distress was an acute asthma exacerbation, and she was treated accordingly with bronchodilators and steroids. However, the patient failed to improve with the corresponding treatments, and other options needed to be considered. Given our patient’s history and hypotonic presentation, the idea of upper airway compromise with impaired airway clearance was explored. This led to the atypical use of Dornase alfa and pulmonary hygiene, which resulted in successful treatment.

Dornase alfa is a recombinant human deoxyribonuclease I that acts as a mucolytic by cleaving DNA found in respiratory secretions. Infected lung secretions contain an increased concentration of extracellular DNA due to the degeneration of neutrophils after they mount an initial immune response towards the source of infection. The excess DNA increases the viscosity of sputum and airway secretions, thus contributing to impaired mucociliary clearance from the lower airways [10]. Dornase alfa hydrolyzes the DNA, liquifying the viscous mucus to allow for improved mucociliary clearance [11]. Although only currently accepted as an effective treatment for cystic fibrosis, Dornase alfa has been shown as an effective treatment for status asthmaticus, atelectasis, chronic sinusitis, primary ciliary dyskinesia, expiratory muscle paralysis, and empyema [12,13]. Although not commonly indicated for pediatric patients presenting with respiratory distress without underlying cystic fibrosis, the unorthodox use of Dornase alfa in our patient led to drastic improvements.

## Conclusions

This case demonstrates a unique case with an atypical management of an infant diagnosed with both PWS and MBS, a rare combination that is lacking representation in literature. The patient presented with respiratory distress as a result of impaired mucociliary clearance secondary to neuromuscular weakness found in both syndromes. When Dornase alfa was used in conjunction with the hypertonic nasal saline, the patient’s rhonchi and tachypnea resolved. By focusing on the possibility of underlying airway hypotonia, our patient’s symptoms were successfully resolved. Therefore, this case demonstrates the importance of physicians maintaining a high index of suspicion to consider uncommon etiologies of respiratory distress, especially in a patient with underlying neuromuscular conditions.
